# Influence of Long-Term Zinc Administration on Spatial Learning and Exploratory Activity in Rats

**DOI:** 10.1007/s12011-015-0597-8

**Published:** 2016-01-06

**Authors:** Agnieszka Piechal, Kamilla Blecharz-Klin, Justyna Pyrzanowska, Ewa Widy-Tyszkiewicz

**Affiliations:** Department of Experimental and Clinical Pharmacology, Centre for Preclinical Research and Technology CePT, Medical University of Warsaw, Banacha 1b, 02-097 Warsaw, Poland

**Keywords:** Zinc supplementation, Spatial memory, Water maze, Hole-board, T-maze

## Abstract

Animal brain contains a significant amount of zinc, which is a cofactor for more than 300 enzymes. Moreover, it provides the basis for functioning of more than 2000 transcription factors, and it is necessary for memory formation and learning processes in the brain. The aim of this study was to investigate the effect of zinc supplementation on behavior in 3-month-old rats. For this purpose, the Morris water maze paradigm, hole-board, and T-maze were used. Wistar rats received a solution of ZnSO_4_ in drinking water at the doses of 16 mg/kg (Zn16 group) and 32 mg/kg (Zn32 group). In rats pretreated with the lower dose of zinc, the improvement of the mean escape latency was observed in comparison to the control group and Zn32 group. During memory task, both ZnSO_4_-supplemented groups showed an increase in crossings over the previous platform position. Furthermore, the exploratory activity in Zn16 group was improved in comparison to Zn32 and control group. In the brains of zinc-supplemented rats, we observed the higher content of zinc, both in the hippocampus and the prefrontal cortex. Hippocampal zinc level correlated positively with the mean annulus crossings of the Zn16 group during the probe trial. These findings show that the long-term administration of ZnS0_4_ can improve learning, spatial memory, and exploratory activity in rats.

**Graphical Abstract:**
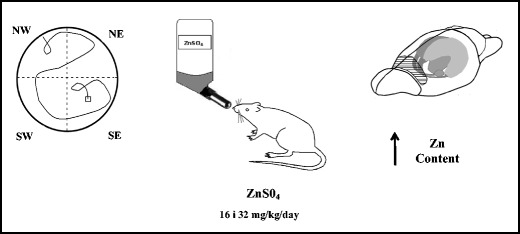
Improvement of spatial learning, memory, and exploratory behavior

Improvement of spatial learning, memory, and exploratory behavior

## Introduction

Zinc, besides iron, is the second most abundant trace element in the body. Iron occurs primarily in the blood, but zinc is the most prevalent metal in the human tissue. The body of a 70-kg man contains approximately 2.3 g of zinc. Zinc is found in all tissues—about 85 % of the whole body’s zinc is present in the bones and muscles. Another 11 % resides in the skin and in the liver. The remaining 2–3 % of zinc is located in other tissues. We can find only 0.1 % of the total zinc in the blood.

Some amount of zinc is found in the central nervous system (CNS). The brain contains about 1.5 % of the total zinc present in the body. Distribution of the metal in the brain is not homogeneous. The higher concentration of zinc can be found rather in the gray than in the white matter, especially in such structures as the cerebellum, cortex, and amygdala. Notwithstanding the highest amount of this trace element is present in the hippocampus, a region that participates in spatial learning and memory. It is expected that Zn^2+^ may be involved in the regulation of hippocampal function as an essential element for synapse formation and structural plasticity [1, 2].

In the CNS, three pools of zinc are described: first, protein-bound zinc acting as a membrane-bound metalloprotein (protein-metal complex pool involved in metal and no-metabolic reactions ∼85 %), second, a vesicular pool located in the synaptic vesicles of the nerve terminals (∼10–15 %), and third, an ionic pool of the free zinc or loosely bound ions in the cytoplasm (not bound to protein) [1]. The average of the total brain zinc concentration is estimated at about 150 μmol/l [3]. Free zinc ion concentration from the cultured neurons comprises approximately 500 nmol in the brain extracellular fluid. On the other hand, the zinc content of the synaptic vesicles is higher than about 1 mmol/l [4, 5]. Zinc is present in the vesicles in co-localization with glutamate. All zinc-containing neurons are glutamatergic, but only certain glutamatergic neurons contain zinc.

Zinc is an essential component of the brain and plays a key role in the development of the CNS during fetal and postnatal life [6]. Zinc-dependent enzymes are involved in cell replication processes necessary for the growth of the brain. Zinc-finger proteins provide framework structure for the brain and are important for neurotransmission in the mossy fiber system of the hippocampus responsible for memory operations. In addition, zinc is involved in metabolic processes outside of the CNS, such as hormones transport and production of neurotransmitter precursors that will eventually affect the brain functions [7]. Moreover, zinc is involved in other biological processes, including the DNA synthesis, normal growth, behavioral responses, brain development, bone formation, and reproduction [8].

Zinc deficiency results in growth retardation, testicular atrophy, skin and mucous membranes changes, poor appetite, delayed wound healing, cell-mediated immune dysfunction, and an abnormal neurosensory responses [9].

It was proposed that zinc deficiency is linked with nervous system disorders, including mental disturbances and loss of sensory acuity [7, 10, 11]. Zinc deprivation may also result in increased stress response as well as altered emotionality and depression-induced behavior (aggression and anxiety) along with reduced attention and deterioration of learning and memory [12–15].

Our previous report showed that zinc supplementation in rats in the prenatal and early postnatal period improves spatial learning, cognitive function, and locomotor activity of the offspring. We also demonstrated the correlation between zinc content in the hippocampus and spatial memory improvement in neonatal rats subjected to supplementation [16]. This study was planned in order to characterize the effect of permanent supplementation of zinc on learning, memory consolidation, and exploratory activity in the 3-month-old rats.

## Materials and Methods

### Animals

Twenty-four 3-month-old male Wistar rats, initially weighing 200–220 g, were used in this study. The rats were housed individually in plastic cages (26 × 42 cm, 18 cm high) with wood cuttings as bedding, under a 12:12 light/dark schedule. During the experiment, the animals had constant access to drinking water and food (Labofeed, Kcynia; concentration of zinc, 210 mg/kg). A solution of zinc sulfate was prepared using tap water (basic Zn content <0.001 mg/ml). Water was analyzed regularly to confirm the concentration of the metal.

All animal testing was conducted according to the Directive 2010/63EU of the European Parliament and of the Council of 22 September 2010 on the protection of animals used for scientific purposes, after approval of the Ethical Committee for Animal Experiments at the Medical University of Warsaw.

### Zinc Treatment

**Fig. 1:**
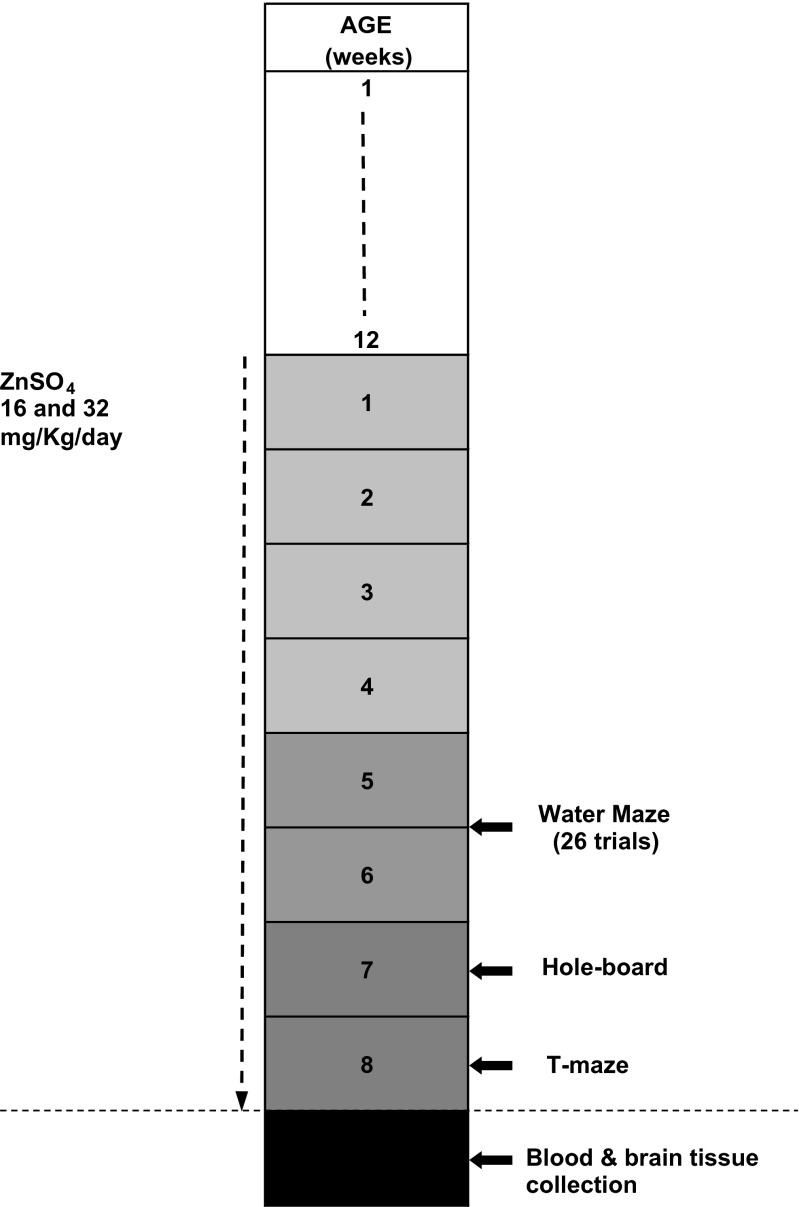
Scheme of zinc treatment and sequence of behavioral tests

In the present study, the effects of zinc supplementation were analyzed in the modified Morris water maze task, hole-board, and T-maze. The animals were divided into three groups and treated as follows: (1) drinking water (Con, *n* = 8); (2) solution of ZnS0_4_ at dose 16 mg/kg b.w. (Zn16, *n* = 8); and (3) solution of ZnS0_4_ at dose 32 mg/kg b.w. (Zn32, *n* = 8). Solutions of zinc were administered orally in the drinking water, and the daily concentration was 16 and 32 mg/kg per rat. Rats were treated for 4 weeks before the experiment and over 4 weeks of behavioral tests (Fig. 1).

### Behavioral Tests

#### Water Maze

Behavioral observations were conducted in the modified version of the Morris water maze test [17]. The experiment took place during the light portion of the cycle between 08:00 a.m. and 03:00 p.m.

In this task, the rats learnt how to discover the location of a transparent platform in the pool. The pool was 1.40 m diameter and 0.50 m high and was filled with 23 °C water (0.30 m). The pool was surrounded by several prominent cues as spatial coordinates. Their positions were not changed during experiment. The pool was divided into four quadrants: northeast (NE), northwest (NW), southeast (SE), and southwest (SW). A submerged plexiglas platform (10 cm × 10 cm) was placed 1 cm under the water. During the acquisition and remainder test, the platform was located in the center of SE quadrant and for the time reversal task in NW quadrant.

For each trial, the animal was put in the water facing the wall of the swimming pool at one of starting the points, excluding the quadrant with the platform. The order of the starting points was randomized and changed every day. The trial was terminated when the rat entered the platform or when 60 s has elapsed. If the animal could not find the platform within this time, it was placed on the platform for 15 s by the experimenter before the next trial was initiated. After completion of the session, the rat was wiped dry and returned to its cage.

During acquisition (days 1–4), remainder (day 8), and transfer (day 9) of the water maze task, all animals had one session consisting of four trials each day. In the memory test (probe trial: days 5 and 10), the platform was removed from the pool, and all rats swam for 60 s.

Water maze test was recorded on videotape. At the same time, the data obtained during the recording were processed by the system Chromotrack, San Diego Instruments. Thus, a precise visual course of the swim distance and the latency to target quadrant to find the submerged platform was calculated. During the memory test, we have analyzed the time spent in each quadrant of the pool and the number of passes over the previous location of the platform. The movements of the rat were sampled at 18 times per second.

#### Hole-Board Test

The hole-board apparatus was used to evaluate exploratory and motor activity in rodents [18, 19]. The device consisted of white wooden, square box, measuring 1 m × 1 m. The walls were 40 cm high, and the top of the box was open for the observer. Sixteen holes (3.8 cm in diameter and 5 cm deep) were cut into the floor of the apparatus. Each hole was located in the middle of 16 square sections (0.25 m × 0.25 m). During 5 consecutive days, each rat was located in the center of the box, back to the experimenter. Each trial lasted 5 min. At the end of the trial, animal was transferred to the home cage. After each trial, the hole-board apparatus was carefully cleaned with a 2 % solution of acetic acid and water.

During each 5-min trial, behavioral data were recorded on the video camera situated above the testing box. The following behavior patterns were taken into account: the number of head-dippings (hiding the head to the eye level into one of the holes), climbings (the animal stands on its back legs and raises its forepaws off the ground, extending its body vertically), crossings (number of square sections entered), and motor activity (total time of movement).

#### T-Maze Test

T-maze is a behavioral test created for measuring spontaneous alternation and motor activity [20]. T-maze apparatus was built from white wooden entrance arm (0.65 m × 0.18 m), two cross arms (0.65 m × 0.18 m), and 0.2 m high walls. In the alternation task, each session consisted of two trials. During the first trial, the rat was located at the top of the entrance arm, and the choice of either of the arms and time of the entry was registered. The trial was terminated when the rat entered with four paws into one of the cross arms. After entering, one of the arms of the rat was removed to its cage. After 60 s, animal was placed again on the starting position, and a second trial was performed. Every day, two sessions, with 4-h intervals, were conducted. The attempt ended if the animal’s four paws went into one of the horizontal arms of the T-maze or if the rat did not turn to any of the arms within 180 s. Between trials, the T-maze was cleaned with an acetic acid solution. The principle of the spontaneous alternation is the phenomenon in which the animal chooses one arm during the first attempt and the second arm in the next attempt. For each rat, an alternation percentage was calculated according to the following formula: number of proper choices divided by quantity of sessions and multiplied by 100. The motor activity was measured as the total latency to choose one of the arms of the T-maze.

### Biochemistry

#### Concentration of Zinc in the Blood

After completion of the behavioral experiments, the rats were decapitated. The blood was poured into the glass tubes and kept in 4 °C temp. After 1 day, serum was collected from the tube and centrifuged at 3000×*g* for 15 min at 4 °C. Then the supernatant was moved to a clean tube and frozen at −80 °C until measurement of zinc in serum was made.

Zinc concentration in the blood samples was determined with the atomic absorption spectrophotometer (Solar, Pey Unicam) as described by Kalinowski et al. [21]. The content of serum zinc was expressed in micrograms per milliliter.

#### Zinc Concentration in the Brain

After the decapitation of the rat, the regional brain concentrations of zinc were estimated in the selected brain regions: the prefrontal cortex and hippocampus. Structures were quickly weighed, frozen, and stored in a deep freezer (−80 °C) until they were assayed. Then, to the tube, 2 ml of concentrated nitric acid (d 1.40 g/ml, JT Baker Analyzed) was added. The samples were microwave digested (Plazmatronika, model SM1) and dissolved in 10 % nitric acid. Zn content was determined by atomic absorption spectrometry (Analyst 300, Perkin Elmer, USA) as described by Kalinowski et al. [21]. Linear calibration was made with 40, 100, and 500 μg/l zinc standards prepared from Spectrascan element standard (1000 ± 5 μg/ml in 2.5 % nitric acid) for atomic spectroscopy, with a zinc content of 10 μg/ml (Teknolab AB, Drøbak, Norway).

The accuracy of determination of zinc was verified using a Quality Control Standard 3 (Cerillant Co., Austin, USA), Bovine Liver reference sample (SRM1577b/Bovine Liver/NIST, Gaithersburg, USA), and as a blank 0.1 M HNO_3_ (Suprapur).

Zinc concentration in the structures of the brain was expressed in micrograms per gram wet tissue.

### Statistical Analysis

All results were presented as mean values ± standard error. To assess differences during acquisition process, ANOVA with repeated measures (treatment × day × trial) was used. All post hoc tests were performed using Newman-Keuls and *T* test to identify any significant differences. Correlation coefficients between learning performance and the level of zinc in the hippocampus and prefrontal cortex were determined using simple linear regression analysis according to Pearson’s *r* correlation. Correlations have been calculated from the results of an individual rat. All the hypotheses tested used a significance level of 0.05.

## Results

### Body Weight

The animals were weighed once a week. There were no significant changes in body weight in rats receiving zinc supplementation for the first 4 weeks [F_(2,21)_ = 0.62, *p* > 0.05]. However, after 4 weeks of zinc administration, we have observed significant reduction of body weight in both groups of rats treated with zinc as compared to control [F_(2,21)_ = 4.31, *p* < 0.05]. This state of affairs remained unchanged until the end of our experiment in the eighth week. Our observations are consistent with other studies, where zinc-treated rats had significantly decreased body weight by reducing energy intake and improving leptin sensitivity after 4 weeks of supplementation [22].

### Water Maze

#### Acquisition Trials (Days 1–4)

**Fig. 2:**
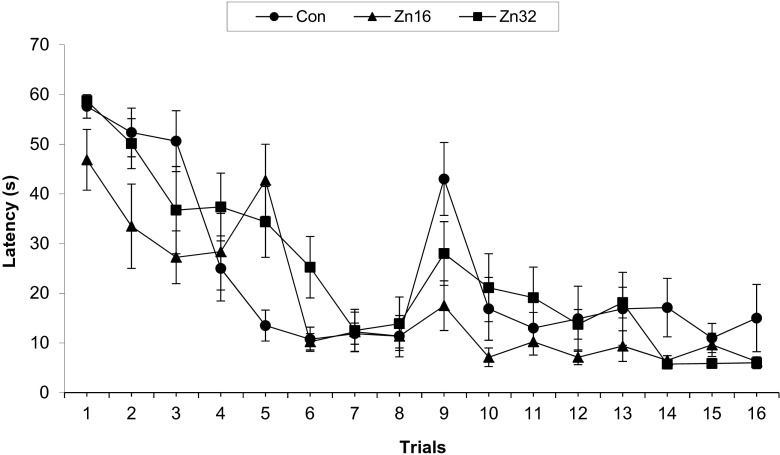
Escape latency (±S.E.) during acquisition of the spatial navigation task (16 trials) for the control and rats supplemented with ZnSO_4_ (Zn16, Zn32)

A repeated ANOVA measures showed a significant mean effect for escape latency, F_(2,21)_ = 6.68, *p* < 0.01. A significant treatment × day interaction, F_(6,63)_ = 3.05, *p* < 0.01, and for treatment × trial, F_(18,189)=_1.9, *p* < 0.05, were found. The Zn16 group (17.89 ± 1.57 s) had significantly shorter mean escape latency over 4 days than the control (23.8 ± 1.9 s) and Zn32 group (24.17 ± 1.8 s) (Newman–Keuls test, *p* < 0.05) (Fig. 2). A significant effect on speed was not observed, F_(2,21)=_0.97, *p* > 0.05 (Con 0.24 ± 0.004 m/s; Zn16 0.26 ± 0.005 m/s; Zn32 0.23 ± 0.005 m/s).

#### The Probe Trial (Day 5)

**Fig. 3:**
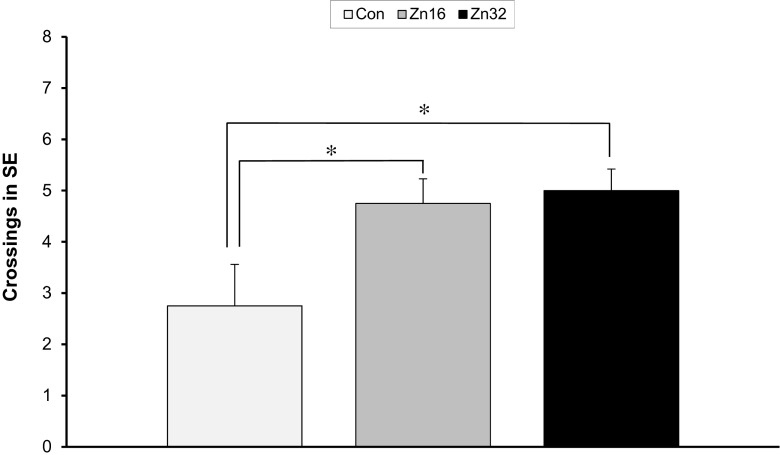
Spatial probe data from the platform area crossings of the control and ZnSO_4_-treated rats in the water maze task on day 5 (trial 17). The test was run in the same manner as the acquisition trials, except that the target was absent, and the trial was terminated after 60 s. The measure given is platform crossings ± SE: the number of times the rat passed through a nominal area defining the originally correct platform position. *p < 0.05

During the memory test, the number of crossings over the previous original platform position in SE quadrant showed differences between groups (F_(2,21)_ = 4.19, *p* < 0.05). The number of annulus crossings was increased for both ZnSO_4_-supplemented groups (Zn16, 4.75 ± 0.48; Zn32, 5.0 ± 0.42) in comparison to the performance of control (Con 2.75 ± 0.81) (Newman–Keuls test, *p* < 0.05) (Fig. 3).

**Fig. 4:**
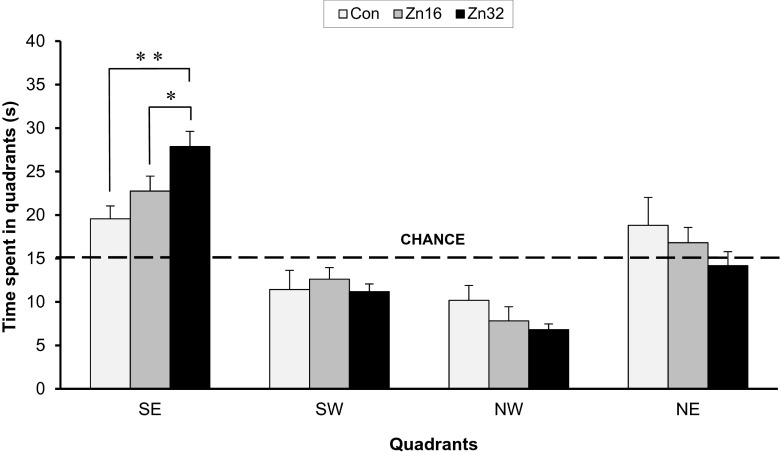
Spatial probe data from the SE quadrant area crossings of the control and ZnSO_4_-treated rats in the water maze task on day 5 (trial 17). The test was run in the same manner as the acquisition trials, except that the target was absent, and the trial was terminated after 60s. *p < 0.05; **p < 0.01

The analysis of variance demonstrated statistical difference between the groups in the percentage of time spent in the target (SE) quadrant in which the platform was situated during acquisition trials, F_(2,21)_ = 6.39, *p* < 0.01. The animal treated with higher dose of zinc spent significantly more time (27.88 ± 1.75 s) in the SE quadrant in comparison to control (19.56 ± 1.48 s) (Newman–Keuls test, *p* < 0.01) and Zn16 group (22.75 ± 1.72 (Newman-Keuls test, *p* < 0.05) (Fig. 4). A significant effect on speed was not observed, F_(2,21)_ = 1.14, *p* > 0.05 (Con 0.26 ± 0.013 m/s; Zn16 0.28 ± 0.01 m/s; Zn32 0.29 ± 0.007 m/s).

#### Remainder Trials (Day 8)

After a 2-day break in swimming, the platform was placed in the SE quadrant, and the training of four trials was resumed for 1 day (the eighth day of the experiment). ANOVA did not show any significant differences in mean escape latency, F_(2,21)_ = 0.46, *p* > 0.05 (Con, 11.31 ± 2.01 s; Zn16, 9.251.43 s; Zn32, 9.4 ± 1.85 s), and swimming speed, F_(2,21)_ = 3.08, *p* > 0.05 (Con, 0.31 ± 0.001 m/s; Zn16, 0.29 ± 0.01 m/s; Zn32, 0.29 ± 0.01 m/s).

#### Transfer Test (Day 9)

On the nineth day, the position of the platform was changed to the opposite quadrant (NW). To compare the crossings over the old quadrant (SE) for each group, data were normalized by the ratio of relative corrected crossings (RCC; the crossing of the trial 22 divided by the escape latency and multiplied by 60). The analysis of variance did not show any significant effects on latency, F_(2,21)_ = 0.69, *p* > 0.05 (Con, 19.34 ± 2.58 s; Zn16, 16.84 ± 3.35 s; Zn32, 20.28 ± 3.28 s), relative corrected crossings, F_(2,21)_ = 0.06, *p* > 0.05 (Con, 5.5 ± 1.78; Zn16, 5.32 ± 0.78; Zn32, 5.95 ± 1.11), and swimming speed, F_(2,21)_ = 0.59, *p* > 0.05 (Con, 0.3 ± 0.009 m/s; Zn16, 0.29 ± 0.009 m/s; Zn32, 0.28 ± 0.006 m/s).

#### The Probe Trial (Day 10)

**Fig. 5:**
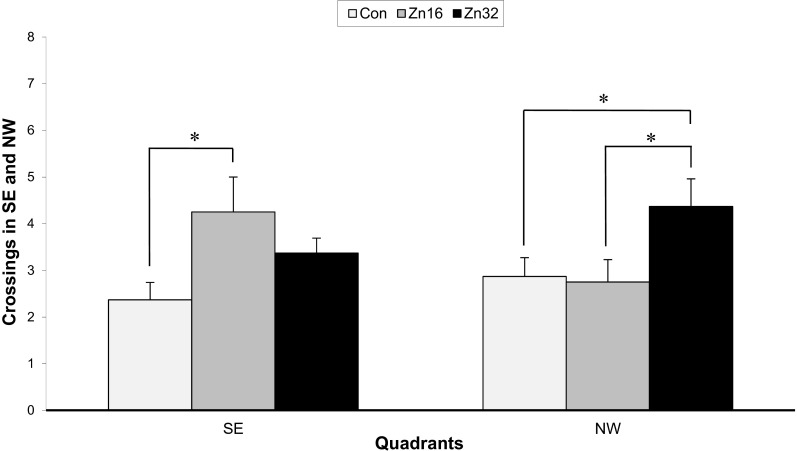
Spatial probe data from the platform area crossings of the control and ZnSO_4_-treated rats in the water maze task on day 10 (trial 26). The target was absent, and the trial was terminated after 60 s. The measure given is platform crossings (over SE and NW) ± SE. *p < 0.05

The number of crossings over the old platform position (SE), F_(2,21)_ = 3.27, *p* < 0.05, like for the new location (NW), F_(2,21)_ = 3.25, *p* < 0.05, was different for particular groups (Fig. 5). The group treated with ZnSO_4_ in a dose of 16 mg/kg b.w./day (Zn16, 4.25 ± 0.75) showed an increased number of crossings in the old position in comparison to the control group (2.37 ± 0.37) and animals pretreated with 32 mg ZnSO_4_ (Zn 3.37 ± 0.32). However, the number of crossings over the new platform position in the NW quadrant increased for Zn32 group (4.37 ± 0.59) compared to Con (2.87 ± 0.4) and Zn16 (2.75 ± 0.48) groups.

The animals treated with ZnSO_4_ spent the same time in SE quadrant (Zn16, 20.5 ± 1.34; Zn32, 18.87 ± 1.22) and in NW quadrant (Z16, 10.62 ± 1.41; Zn32, 12.5 ± 0.82) as did the control group (SE: Con, 19.25 ± 0.96; NW: Con, 11.25 ± 0.59).

#### Hole-Board Results

The effect of ZnSO_4_ pretreatment on exploratory behavior of rats in the hole-board test is shown in Table 1.

**Table 1:** Results of the hole-board test (days 1–5) in the control (*n* = 8) and zinc-supplemented rats (Zn16, *n* = 8 and Zn32, *n* = 8). Data are presented as mean ± SE levels

Parameters of exploratory behavior (mean ± SE)				
	Motor activity (s)	Head-dipping (*n*)	Climbing (*n*)	Crossing (*n*)
Con	27.55 ± 4.8	2.97 ± 0.43	2.57 ± 0.37	2.8 ± 0.24
Zn16	43.42 ± 5.0^▲^	5.0 ± 0.54*	5.3 ± 0.51**	4.97 ± 0.78
Zn32	30.3 ± 3.88	3.27 ± 0.45^#^	3.15 ± 0.34^##^	3.37 ± 0.23

*Zn16 vs Con, *p* < 0.05; **Zn16 vs Con, *p* < 0.01 (Newman–Keuls test)

^#^Zn16 vs Zn32, *p* < 0.05; ^##^Zn16 vs Zn32, *p* < 0.01 (Newman–Keuls test)

^▲^Zn16 vs Control, *p* < 0.05 (*T* test)

In the hole-board test, the values of mean head-dipping counts were different among the groups, F_(2,21)_ = 4.65, *p* < 0.05. There was significant group × day interaction, F_(8,84)_ = 2.71, *p* < 0.01. Post hoc analysis showed an increase of head-dipping count in Zn16 group versus control animals (*p* < 0.05, Newman-Keuls test).

ANOVA demonstrated statistically significant differences between mean climbing count, F_(2,21)_ = 11.075, *p* < 0.001. It was significant for the day of training, F_(4,84)_ = 4.08, and group × day interaction, F_(8,84)_ = 3.87, *p* < 0.001. ZnS0_4_ at dose 16 mg/kg/day significantly increased climbing behaviors compared to the control and Zn32 group (*p* < 0.01, Newman–Keuls test).

ANOVA demonstrated significant treatment effects on motor activity, F_(2.21)_ = 3.36, *p* < 0.05. It was significant for the group × day interaction, F_(8,84)_ = 2.3, *p* < 0.05. Further analysis showed a significant increase in the motor activity in Zn16 group compared to control (*p* < 0.05, *T* test).

There was no significant differences in the mean number of crossing count: F_(2,21)_ = 2.05, p > 0.05.

#### T-Maze Results

**Fig. 6:**
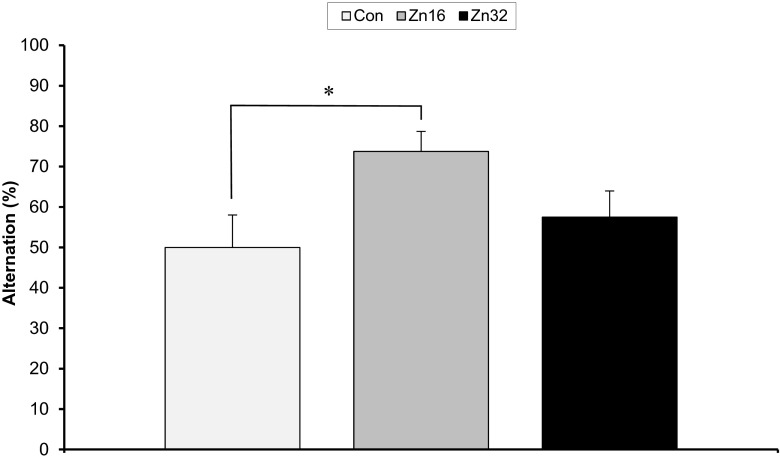
T-maze spontaneous alternation (±SE) average of all trials for the control (*n* = 8) and zinc-supplemented rats (Zn16, *n* = 8; Zn32, *n* = 8). *p < 0.05

**Fig. 7:**
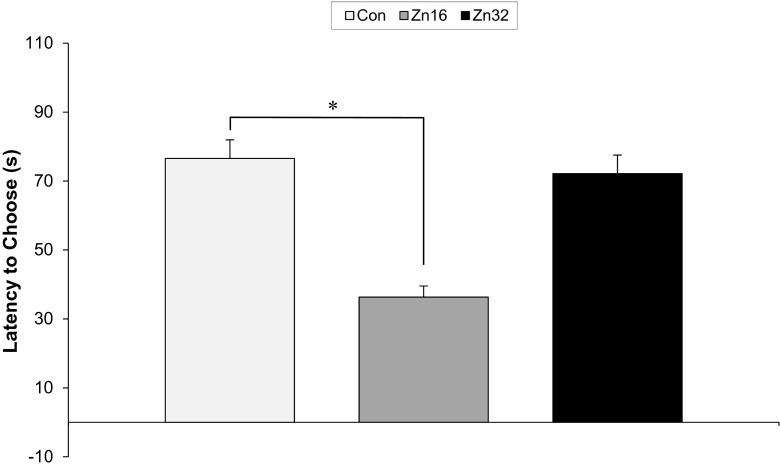
Latency to choose goal arm for the control (*n* = 8) and zinc-supplemented rats (Zn16, *n* = 8; Zn32, *n* = 8). *p < 0.05

The values of mean alternation percentage were different among the groups, F_(2,21)_ = 3.37, *p* < 0.05. Spontaneous alternation behavior was significantly higher in rats pretreated with Zn16 compared to control and Zn32 group (*p* < 0.05, Newman-Keuls test) (Fig. 6). *T* test analysis of latencies to choose a proper arm showed the mean latency for the Zn16 group (36.33 ± 3.22 s) to be significantly smaller than both Zn32 (72.16 ± 5.38 s) and the control group (76.55 ± 5.43 s) (Fig. 7).

#### Serum Zinc Level

There was a significant difference in the concentration of zinc in the blood from rats treated with zinc. Rats that received ZnSO_4_ at a dose of 32 mg/kg/day showed higher blood levels of zinc in comparison to the control group (*p* < 0.05, *T* test) (Table 2).

**Table 2:** Brain tissue and serum zinc level in the control (*n* = 8) and Zn-supplemented rats (Zn16, *n* = 8; Zn32, *n* = 8). Data are presented as μg/g (mean ± SE)

Zn level in the brain and serum μg/g wet tissue (mean ± SE)			
	Prefrontal cortex	Hippocampus	Serum
Con (*n* = 8)	15.36 ± 0.11	15.27 ± 0.14	1.06 ± 0.03
Zn16 (*n* = 8)	16.16 ± 0.25*	17.05 ± 0.45*	1.13 ± 0.02
Zn32 (*n* = 8)	16.26 ± 0.25*	17.68 ± 0.87*	1.16 ± 0.02^▲^

^▲^Zn32 vs Control, *p* < 0.05 (*T* test)

*Zn16 and Zn32 vs Control, *p* < 0.05 (Newman–Keuls test)

#### Hippocampal and Cortical Zinc Level

ANOVA showed significant differences in the level of zinc in the prefrontal cortex (F_(2,21)_ = 5.19, *p* < 0.05) and hippocampus (F_(2,21)_ = 4.69, *p* < 0.5). Higher concentration of zinc was observed in rats pretreated with zinc sulfate compared to the control group (*p* < 0.05, Newman–Keuls test) (Table 2).

#### Correlation Between Zinc Levels and Spatial Memory

**Fig. 8:**
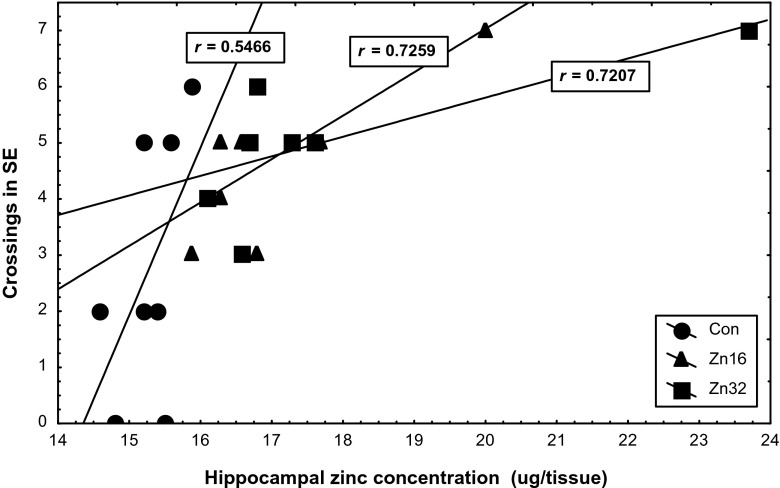
Correlations between the number of crossings over the position of a hidden platform in the memory test on the fifth day of the experiment and the zinc concentration in the hippocampus in control and zinc-supplemented rats. *r* indicates Pearson’s correlation coefficient

A measure of spatial learning accuracy was used to estimate whether brain levels of zinc were connected with the cognitive ability in the zinc-supplemented rats. Hippocampal zinc levels correlated positively with the mean annulus crossing of the Zn16 (*r* = 0.7259, F_(1,6)_ = 6.68, *p* < 0.05) and Zn32 (*r* = 0.7207, F_(1,6)_ = 6.48, *p* < 0.05) group during the probe trial (Fig. 8). There was no correlation between the zinc contents in prefrontal cortex and results of the probe trial in all tested groups.

## Discussion

Zinc performs many important functions in all living creatures (plants, animals, and humans) and is present in all cells of the human body. Elevated concentration of zinc occurs in the CNS, within neurons and glial cells, where zinc is engaged in synaptic neurotransmission. Particularly high content of zinc is present in the hippocampus, the structure involved in learning and memory. However, the role of zinc in cognitive processes is not fully understood.

Effects of zinc supplementation on cognitive processes are described in numerous publications. Yet, the results are still inconclusive. In this study, we discuss the findings consistent with our previous study, indicating that the administration of the zinc throughout the prenatal and in the early postnatal period improves the cognitive skills [16]. In experiments parallel to ours, Tahmasebi Boroujeni et al. [23] demonstrated that the rats supplemented with zinc had a significant increase of time spent in the target quadrant, what confirms a better spatial memory consolidation. Moreover, the studies of Yu et al. [24] showed that zinc supplementation after delivery reversed impaired memory and abnormal ultrastructure of neurons in rats deprived of zinc during pregnancy.

In this publication, we describe a positive correlation between the metal content in the hippocampus and spatial memory in rats from Zn16 group as well. In our previous study on rat pups, we showed that the number of crossings over the platform in a memory test was positively correlated with the concentration of zinc in the hippocampus [16]. This effect appeared for the lower and the higher dose of the metal. However, the correlation was more pronounced in the group receiving the lower dose of zinc. It can be assumed that the lower dose is optimal for the improvement of learning and memory consolidation. Furthermore, in the abovementioned group of rats treated with the lower dose (Zn16), the increased locomotor activity was seen. The study demonstrated a dose-dependent effect of zinc on locomotor activity and the memory outcome. From here, it can be suggested that the dose response is represented by a curve in the shape of an inverted U letter. The biphasic response indicates that small doses evoke stimulation of the phenomenon studied, and the higher dose leads to the inhibition of the observed effects. Zinc concentration dose-dependently exhibits protective or excitotoxic effect. It is very likely that the different effect of zinc is due to variation in the sensitivity of cells to excitotoxicity in each area. Neuroprotective effect is observed when the zinc is in the form of chelates [25].

Similar dose-dependent activity of zinc in memory processes was also described in the literature of the subject. Mice fed with high doses of zinc revealed hippocampus-dependent memory dysfunction. In contrast, mice supplemented with a low dose of zinc displayed better differentiation between two similar chambers in a T-maze [26]. Moazedi et al. [27] demonstrated that the consumption of zinc (30 mg/kg/day) for 2 weeks during pregnancy may more effectively affect working memory of the offspring than other doses (20, 50, 70, 100 mg/kg/day). The high dose (100 mg/kg/day) induced significant impairment in working (short-term) memory, and there were no significant differences in long-term memory for the zinc-supplemented animals. In contradiction with our results, Flinn et al. [28] found memory deficits after increased consumption of the zinc, both in pregnancy and in adulthood in the zinc-treated rats. However, unlike our research, those animals received a higher dose of zinc for a longer period of time (3 and 9 months). Also, Chrosniak et al. [29] showed that regularly administered zinc may effect on cognitive deficits. When rats were given zinc carbonate with cuprous chloride in drinking water, spatial memory deficit was not seen. Such results indicate that the addition of copper may remediate certain deficiency seen in high dose zinc-treated rats.

Zinc—inextricably linked with the synaptic vesicles—appears to modulate a number of postsynaptic receptors. We know that the pharmacology of the NMDA receptor in the early postnatal period is different from that in adults. The expression of the NR_1_ and NR_2A_ subunits increases with age and peaks in the 21st day of life. In adult rats, NR_1_/NR_2B_ complex dominates and may explain different effects of zinc in pups and 3-month-old rats [30].

In the adult hippocampus, the formation of new neurons or neurogenesis still remains active [31]. Corniola et al. [32] hypothesized that zinc plays a role in the adult hippocampal neurogenesis by regulating the p53-dependent molecular mechanisms that controls progenitor cell proliferation and survival of neurons. It is likely that the beneficial effect of zinc supplementation on rats observed in our study may be the result of neurogenesis in the hippocampus.

Zn^2+^ is also essential for synaptogenesis and synapse maturation as its deficiency impairs neurogenesis and development of proliferative neuronal precursors. Its shortage impairs developmental neurogenesis through the reduction of neuronal precursors of proliferation [2, 33]. Spatial overtraining in a water maze could induce synaptogenesis in the CA_3_*stratum oriens* region. However, this phenomenon has been observed only for the duration of the 3-day experiment. Animals trained only for 1 day did not show increments in mossy fiber terminals [34]. It can be assumed that in our prolonged experiment during the 10-day water maze procedure, synaptogenesis could occur in the hippocampus of the rats.

The role of zinc in the exploratory behavior is poorly understood. In the study using open field, Sobhanirad et al. [35] found that high dose of zinc methionine increased both the exploratory and locomotor activity in rats. In our study, the lower dose of zinc resulted in an increase of the number of head-dippings and climbings. However, Tahmasebi Boroujeni [23] did not show any effect on motor activity in animals supplemented with zinc. In another experiment, Takeda et al. [36] studied the exploratory behavior during novelty stress. They showed that locomotor activity was the highest in the first period of 10 min of the test and decreased over time. In their experiment, extracellular zinc was decreased on days 1 and 2 and did not return to the baseline at day 8. On days 1 and 2, the level of extracellular glutamate in the hippocampus significantly increased during exploratory behavior. It may represent habituation to the novel environment. This suggests that an increased intake of zinc is involved in the cognitive processes.

In the present study, we demonstrated that supplementation increases the zinc level both in the hippocampus and in the prefrontal cortex. Sowa-Kućma et al. [37] also showed that zinc administration increases the concentration of zinc in these tissues. The authors revealed an increase in presynaptic and extracellular levels of zinc in both structures. They postulated the antidepressant role of increased concentration of zinc observed in the prefrontal cortex after imipramine and citalopram administration. The results of our experiments in the hole-board and the T-maze with a significant increase in the locomotor activity after zinc supplementation may as well indicate antidepressant properties of the metal.

Guidolin et al. [38] showed the significant decrease in the amount of zinc within hippocampal mossy fibers in 24-month-old rats with memory impairment compared to the 5-month-old and non-impaired rats. According to Flinn et al. [28], the brain levels of zinc were increased by the addition of ZnCO_3_ to the drinking water but long-term dietary administration of zinc could lead to cognitive disorders. The results different from our experience were observed by Takeda et al. [39]. They claimed that the object recognition memory deficits may be linked to the preferential increase in Zn^2+^ in CA_1_ pyramidal neurons.

Alterations in the amount of zinc in the hippocampus may be the result of various tests carried before the measurement. It is likely that the change of zinc content in the brain may be due to reduced ion permeability of the blood barrier. The activity of the hypothalamic–pituitary-adrenal (HPA) axis, which is increased by stress and aging, may result in the modification of synaptic Zn, and then alteration in learning and memory processes [40].

In the water maze test, we demonstrated that both doses of zinc improve spatial memory on the fifth and tenth day of the experiment. On the tenth day of the experiment, working memory was enhanced at a higher dosage of zinc. It is difficult to explain this phenomenon. Both doses increase the concentration of the metal in the hippocampus, the structure involved in the spatial memory and in the prefrontal cortex, which is responsible for working memory. Both the “old” and the “new” memories were tested during the tenth day of the experiment. When testing both types of memory, it may come to overlapping of stimuli received by rats and then higher dose prevails. In our experiment, only the higher dose of the metal could improve working memory in animals.

The effect of zinc on the spatial and working memory may be dose dependent. Karami et al. [41] demonstrated that zinc supplementation of lactating rats at a dose of 70 mg/kg/day and less does not affect the reference and working memory. In contrast, zinc administered in 100 mg/kg/day results in deterioration of working memory.

Studies on the impact of the effect of zinc on cognitive processes on humans are also not clear. The ZENITH study revealed that zinc supplementation improves spatial working memory and cognitive function in healthy adults [42]. Furthermore, zinc supplementation in malnourished children improves their developmental quotients, patterns of activity, and neuropsychological functions [43]. However, Taneja et al. [44] demonstrated that after 4 months of zinc supplementation, childrens’ intellectual development index was similar in the intervention and placebo groups. A recent meta-analysis of 18 clinical studies showed no effect of zinc supplementation on cognitive function [45]. The authors argue that there should be more high-quality clinical trials to assess the exact role of zinc supplementation on the development of executive and motor functions. Other recent studies showed a correlation between the serum levels of zinc and cognitive abilities and depression symptoms [46]. Aged patients with lower concentrations of the metal presented certain disorders of memory and symptoms of depression.

## Conclusions

In this study, it was shown that the administration of zinc in rats improves spatial learning, memory, and exploratory behavior. Our current findings are consistent with the reports of cognitive impairment caused by nutritional zinc deficiency either in humans or in animals as well as with an improvement in learning and memory after supplementation. Certainly, beneficial effects of zinc are dose dependent, as we demonstrated in our experiment. The demand for zinc in growing organism is changeable. Of particular importance is the U-shaped dose response for zinc, when toxic effects are related to the presence of either too much or too little zinc. Hence, additional studies are necessary to determine the mechanisms of zinc influence on cognitive development in young subjects.

## References

[1] Frederickson CJ (1989). Neurobiology of zinc and zinc-containing neurons. Int Rev Neurobiol.

[2] Grabrucker AM, Knight MJ, Proepper C, Bockmann J, Joubert M, Rowan M, Nienhaus GU, Garner CC, Bowie JU, Kreutz MR, Gundelfinger ED, Boeckers TM (2011). Concerted action of zinc and ProSAP/Shank in synaptogenesis and synapse maturation. EMBO J.

[3] Takeda A (2000). Movement of zinc and its functional significance in the brain. Brain Res Brain Res Rev.

[4] Weiss JH, Sensi SL, Koh JY (2000). Zn(2+): a novel ionic mediator of neural injury in brain disease. Trends Pharmacol Sci.

[5] Frederickson CJ, Suh SW, Silva D, Frederickson CJ, Thompson RB (2000). Importance of zinc in the central nervous system: the zinc-containing neuron. J Nutr.

[6] Caulfield LE, Zavaleta N, Shankar AH, Merialdi M (1998). Potential contribution of maternal zinc supplementation during pregnancy to maternal and child survival. Am J Clin Nutr.

[7] Golub MS, Keen CL, Gershwin ME, Hendrickx AG (1995). Developmental zinc deficiency and behavior. J Nutr.

[8] Prasad AS (2014). Impact of the discovery of human zinc deficiency on health. J Trace Elem Med Biol.

[9] Prasad AS (2013). Discovery of human zinc deficiency: its impact on human health and disease. Adv Nutr.

[10] Black MM (1998). Zinc deficiency and child development. Am J Clin Nutr.

[11] Halas ES, Eberhardt MJ, Diers MA, Sandstead HH (1983). Learning and memory impairment in adult rats due to severe zinc deficiency during lactation. Physiol Behav.

[12] Peters DP (1978). Effects of prenatal nutritional deficiency on affiliation and aggression in rats. Physiol Behav.

[13] Młyniec K, Davies CL, de Agüero Sánchez IG, Pytka K, Budziszewska B, Nowak G (2014). Essential elements in depression and anxiety. Part I. Pharmacol Rep.

[14] Whittle N, Lubec G, Singewald N (2009). Zinc deficiency induces enhanced depression-like behaviour and altered limbic activation reversed by antidepressant treatment in mice. Amino Acids.

[15] Hagmeyer S, Haderspeck JC, Grabrucker AM, Hagmeyer S, Haderspeck JC, Grabrucker AM (2014). Behavioral impairments in animal models for zinc deficiency. Front Behav Neurosci.

[16] Piechal A, Blecharz-Klin K, Pyrzanowska J, Widy-Tyszkiewicz E (2012). Maternal zinc supplementation improves spatial memory in rat pups. Biol Trace Elem Res.

[17] Widy-Tyszkiewicz E, Scheel-Kruger J, Christensen AV (1993). Spatial navigation learning in spontaneously hypertensive, renal hypertensive and normotensive Wistar rats. Behav Brain Res.

[18] File SE, Wardill AG (1975). Validity of head-dipping as a measure of exploration in a modified hole-board. Psychopharmacologia.

[19] File SE, Wardill AG (1975). The reliability of the hole-board apparatus. Psychopharmacologia.

[20] Yadin E, Friedman E, Bridger WH (1991). Spontaneous alternation behavior: an animal model for obsessive-compulsive disorder?. Pharmacol Biochem Behav.

[21] Kalinowski M, Wolf G, Markefski M (1983). Concentration and subcellular localization of zinc in the hippocampal formation, cerebellum, and whole brain during the postnatal development of the rat. Acta Histochem.

[22] Song MK, Rosenthal MJ, Song AM, Uyemura K, Yang H, Ament ME, Yamaguchi DT, Cornford EM (2009). Body weight reduction in rats by oral treatment with zinc plus cyclo-(his-pro). Br J Pharmacol.

[23] Tahmasebi Boroujeni S, Naghdi N, Shahbazi M, Farrokhi A, Bagherzadeh F, Kazemnejad A, Javadian M (2009). The effect of severe zinc deficiency and zinc supplement on spatial learning and memory. Biol Trace Elem Res.

[24] Yu X, Jin L, Zhang X, Yu X (2013). Effects of maternal mild zinc deficiency and zinc supplementation in offspring on spatial memory and hippocampal neuronal ultrastructural changes. Nutrition.

[25] Frederickson CJ, Maret W, Cuajungco MP (2004). Zinc and excitotoxic brain injury: a new model. Neuroscientist.

[26] Yang Y, Jing XP, Zhang SP, Gu RX, Tang FX, Wang XL, Xiong Y, Qiu M, Sun XY, Ke D, Wang JZ, Liu R (2013). High dose zinc supplementation induces hippocampal zinc deficiency and memory impairment with inhibition of BDNF signaling. PLoS One.

[27] Moazedi AA, Ghotbeddin Z, Parham GH (2007). Comparison of the effects of dose-dependent zinc chloride on short-term and long-term memory in young male rats. Pak J Biol Sci.

[28] Flinn JM, Hunter D, Linkous DH, Lanzirotti A, Smith LN, Brightwell J, Jones BF (2005). Enhanced zinc consumption causes memory deficits and increased brain levels of zinc. Physiol Behav.

[29] Chrosniak LD, Smith LN, Flinn JM, McDonald C, Jones BF (2006). Effects of enhanced zinc and copper in drinking water on spatial memory and fear conditioning. J Geochem Explor.

[30] Sircar R (2000). Developmental maturation of the N-methyl-D-aspartic acid receptor channel complex in postnatal rat brain. Int J Dev Neurosci.

[31] Stangl D, Thuret S (2009). Impact of diet on adult hippocampal neurogenesis. Genes Nutr.

[32] Corniola RS, Tassabehji NM, Hare J, Sharma G, Levenson CW (2008). Zinc deficiency impairs neuronal precursor cell proliferation and induces apoptosis via p53-mediated mechanisms. Brain Res.

[33] Levenson CW, Morris D (2011). Zinc and neurogenesis: making new neurons from development to adulthood. Adv Nutr.

[34] Ramírez-Amaya V, Escobar ML, Chao V, Bermúdez-Rattoni F (1999). Synaptogenesis of mossy fibers induced by spatial water maze overtraining. Hippocampus.

[35] Sobhanirad S, Valizadeh R, Moghimi A, Tahmasebi A (2008). Assessment of changes in anxiety and exploratory behaviors following zinc supplementation in rats. Res J Biol Sci.

[36] Takeda A, Sakurada N, Kanno S, Minami A, Oku N (2006). Response of extracellular zinc in the ventral hippocampus against novelty stress. J Neurochem.

[37] Sowa-Kućma M, Kowalska M, Szlósarczyk M, Gołembiowska K, Opoka W, Baś B, Pilc A, Nowak G (2011). Chronic treatment with zinc and antidepressants induces enhancement of presynaptic/extracellular zinc concentration in the rat prefrontal cortex. Amino Acids.

[38] Guidolin D, Polato P, Venturin G, Zanotti A, Mocchegiani E, Fabris N, Nunzi MG (1992). Correlation between zinc level in hippocampal mossy fibers and spatial memory in aged rats. Ann N Y Acad Sci.

[39] Takeda A, Takada S, Nakamura M, Suzuki M, Tamano H, Ando M, Oku N (2011). Transient increase in Zn2+ in hippocampal CA1 pyramidal neurons causes reversible memory deficit. PLoS One.

[40] Takeda A, Tamano H (2010). Zinc signaling through glucocorticoid and glutamate signaling in stressful circumstances. J Neurosci Res.

[41] Karami M, EhsaniVostacolaee S, Moazedi AA, Nosrati A (2013). The effect of zinc supplementation of lactating rats on short-term and long-term memory of their male offspring. Health Promot Perspect.

[42] Maylor EA, Simpson EE, Secker DL, Meunier N, Andriollo-Sanchez M, Polito A, Stewart-Knox B, McConville C, O’Connor JM, Coudray C (2006). Effects of zinc supplementation on cognitive function in healthy middle-aged and older adults: the ZENITH study. Br J Nutr.

[43] Gardner JM, Powell CA, Baker-Henningham H, Walker SP, Cole TJ, Grantham-McGregor SM (2005). Zinc supplementation and psychosocial stimulation: effects on the development of undernourished Jamaican children. Am J Clin Nutr.

[44] Taneja S, Bhandari N, Bahl R, Bhan MK (2005). Impact of zinc supplementation on mental and psychomotor scores of children aged 12 to 18 months: a randomized, double-blind trial. J Pediatr.

[45] Warthon-Medina M, Moran VH, Stammers AL, Dillon S, Qualter P, Nissensohn M, Serra-Majem L, Lowe NM (2015). Zinc intake, status and indices of cognitive function in adults and children: a systematic review and meta-analysis. Eur J Clin Nutr.

[46] Markiewicz-Żukowska R, Gutowska A, Borawska MH (2015). Serum zinc concentrations correlate with mental and physical status of nursing home residents. PLoS One.

